# Understanding the uptake of diagnostics for sustainable gastrointestinal nematode control by European dairy cattle farmers: a multi-country cross-sectional study[Fn FN1]

**DOI:** 10.1051/parasite/2023002

**Published:** 2023-02-10

**Authors:** Fiona Vande Velde, Lisbeth Hektoen, Claire J. Phythian, Laura Rinaldi, Antonio Bosco, Barbara Hinney, Martin Gehringer, Christina Strube, Katharina May, Gabriela Knubben-Schweizer, Oliva M.D. Martins, Teresa L. Mateus, Violeta-Elena Simion, Johannes Charlier, David J. Bartley, Edwin Claerebout

**Affiliations:** 1 Laboratory for Parasitology, Faculty of Veterinary Medicine, Ghent University Salisburylaan 133 9820 Merelbeke Belgium; 2 Department of Public Health Science, Faculty of Landscape and Society, Norwegian University of Life Sciences Fredrik A. Dahls vei 15 1430 Ås Norway; 3 Department of Production Animal Clinical Sciences, Faculty of Veterinary Medicine, Norwegian University of Life Sciences, 1 Veterinærbygningen Elizabeth Stephansens vei 15 1430 Ås Norway; 4 Department of Veterinary Medicine and Animal Production, University of Naples Federico II, CREMOPAR Via Federico Delpino 1 80137 Naples Italy; 5 Institute of Parasitology, Department of Pathobiology, Vetmeduni Veterinärplatz 1 1210 Vienna Austria; 6 LKV Lower Austria Pater Werner Deibl-Straße 4 3910 Zwettl Austria; 7 Institute for Parasitology, Centre for Infection Medicine, University of Veterinary Medicine Hannover Buenteweg 17 30559 Hanover Germany; 8 Institute of Animal Breeding and Genetics, Justus-Liebig-University of Gießen Frankfurter Str. 94 35392 Giessen Germany; 9 Clinic for Ruminants with Ambulatory and Herd Health Services, Ludwig-Maximilians Universität München Sonnenstrasse 16 85764 Oberschleissheim Germany; 10 Instituto Politécnico de Bragança, Campus de Santa Apolónia 5300-253 Bragança Portugal; 11 CISAS - Center for Research and Development in Agrifood Systems and Sustainability, Escola Superior Agrária, Instituto Politécnico de Viana do Castelo, Rua Escola Industrial e Comercial de Nun’Àlvares 4900-347 Viana do Castelo Portugal; 12 Veterinary and Animal Research Centre (CECAV), UTAD, Associate Laboratory for Animal and Veterinary Sciences (AL4AnimalS) Quinta de Prados 5000-801 Vila Real Portugal; 13 Faculty of Veterinary Medicine, Spiru Haret University Bdul Basarabia 256, Sector 3 030352 Romania; 14 Kreavet Hendrik Mertensstraat 17 9150 Kruibeke Belgium; 15 The Moredun Research Institute, Pentlands Science Park Penicuik EH26 0PZ United Kingdom

**Keywords:** Dairy cattle, Anthelmintic resistance, Decision-making, Farmer behaviour, Multicentre study, Structural equation modelling

## Abstract

To mitigate emerging anthelmintic resistance (AR) in cattle, sustainable gastrointestinal nematode control strategies should be adopted. A multi-centre study was set up to understand the factors affecting European dairy cattle farmers’ adoption of diagnostics and to gauge for differences between regions. The data were collected through a multi-lingual survey by participating countries of the European Co-operation in Science and Technology (COST) action COMbatting Anthelmintic Resistance in ruminants (COMBAR). Four countries provided sufficient data to be included in the data analysis: Norway, Italy, Germany and Austria. Three models were estimated and validated through structural equation modelling. Norway, along with Germany and Austria (pooled dataset) showed similar trends that align with previous studies. AR risk perception had no influence on the adoption intention of diagnostics, a positive influence was found for attitude towards diagnostics and subjective norms (i.e., perceived opinion of others), and a negative influence of attitudes towards anthelminthics. Additionally, routine (i.e., perception of the current treatment) had an indirect effect on adoption intention through attitudes. Italy’s data deviated from these findings, presenting a positive effect of the perceived severity of AR, and perceived behavioural control (i.e., perceived ability to perform a specific behaviour) on adoption intention of diagnostics. Finally, Norway’s data set allowed for inclusion of a measurement of current behaviour in the model, identifying a direct positive effect of the perceived actual behaviour of other farmers on their own behaviour.

## Abstract (fr)

Pour atténuer la résistance émergente aux anthelminthiques (RA) chez les bovins, des stratégies durables de contrôle des nématodes gastro-intestinaux devraient être adoptées. Une étude multicentrique a été réalisée pour comprendre les facteurs affectant l’adoption des diagnostics par les éleveurs de bovins laitiers européens, et pour évaluer les différences entre les régions. Les données ont été recueillies au moyen d’une enquête multilingue menée par les pays participants à l’action de European Co-operation in Science and Technology (COST) COMbatting Anthelmintic Resistance in ruminants (COMBAR). Quatre pays ont collecté suffisamment de données pour être inclus dans l’analyse statistique : la Norvège, l’Italie, l’Allemagne et l’Autriche. Trois modèles ont été estimés et validés par modélisation d’équations structurelles. La Norvège, et d’autre part l’Allemagne et l’Autriche (données regroupées) ont montré des tendances similaires qui correspondent aux études précédentes. La perception de la RA n’avait aucune influence sur l’intention d’adoption des diagnostics, une influence positive a été trouvée pour l’attitude envers les diagnostics et les normes subjectives (c’est-à-dire l’opinion perçue des autres), et une influence négative des attitudes envers les anthelminthiques. De plus, la routine (c’est-à-dire la perception du traitement actuel) a eu un effet indirect sur l’intention d’adoption par le biais des attitudes. Les données de l’Italie s’écartaient des résultats précédents, présentant un effet positif de la gravité perçue du risque de la RA, et du contrôle comportemental perçu (c’est-à-dire la capacité perçue à adopter un comportement spécifique) sur l’intention d’adoption de diagnostic. Enfin, l’ensemble de données de la Norvège a permis d’inclure une mesure du comportement actuel dans le modèle, identifiant un effet positif direct du comportement réel perçu des autres agriculteurs sur son propre comportement.


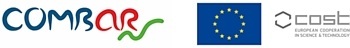


**Special Issue – Combatting Anthelmintic resistance in ruminants**

**Invited Editors: Johannes Charlier, Hervé Hoste, and Smaragda Sotiraki**

## Introduction

Gastrointestinal nematode infections are a common constraint in pasture-based dairy cattle herds and cause a decrease in animal health and wellbeing, productivity and farm profitability [8, 9, 12]. Control practices to prevent production losses due to gastrointestinal nematode infections in livestock depend largely on the use of anthelmintic compounds [24, 42]. However, due to the continued use of these compounds, the industry is increasingly confronted with anthelmintic resistant nematode populations [35]. These findings emphasize the need for sustainable treatment approaches, such as implementation of diagnostic techniques (e.g. faecal egg counts, serum pepsinogen levels, and bulk-tank milk ELISA) to inform treatment decisions. The uptake of methods for sustainable worm control would reduce excessive anthelmintic use [10] and minimize the selection pressure and spread of anthelmintic resistance (AR). In contrast to transboundary, epidemic or zoonotic diseases for which control measures are mostly taken by policy interventions, the control of gastrointestinal nematode infections has remained the individual responsibility of the farmer [11]. Accordingly, to successfully implement sustainable control strategies and truly embed them in common worm management, farmers’ decision-making should be a central point of focus in research and practice.

It is widely accepted that farmers’ decision-making varies, influenced by factors that are not exclusively based on economic considerations or policy [6]. Variability can be explained by a wide range of individual farmer traits (e.g., personality, attitudes, beliefs, intentions, values, skills, and knowledge) [32]. These personal traits often explain more variation in farm performance than farmers’ measurable management practices [44]. To account for these factors, different theoretical frameworks have been applied, to examine a wide range of cattle farmers’ health-related behaviours, such as the control of mastitis [21, 22], Johne’s disease [3, 33], foot-and-mouth disease [15], lameness [27, 28], the implementation of on-farm biosecurity [43], vaccination strategies [41], antimicrobial usage [23, 26] and psoroptic mange [29, 30]. The combination of socio-psychological theories and methodologies with traditional epidemiologic approaches has proven useful for exploring cattle farmers’ intentions and behaviours. The two most commonly used theories are the Theory of Planned Behaviour (TPB) [1] and the Health Belief Model (HBM) [37]. The TPB describes the intention to perform a behaviour as a function of the individuals’ attitude towards the behaviour, their perceived control over whether or not they perform the behaviour and peer/societal influences. The HBM shares common elements with the TPB, but includes potential barriers to performing a behaviour, and the evaluation of potential risk, as well as cues-to-action.

Previous work on control practices against gastrointestinal nematode infections conceptualized both theories to identify several socio-psychological factors that influence dairy cattle farmers’ intention to adopt diagnostic methods [48]. The results showed that farmers’ positive attitude towards diagnostics and the perceived pressure of important referents (e.g., veterinarians, peers) were the main drivers of this intention. Similar results were obtained from a study on the control of psoroptic mange in beef cattle. Adoption of sustainable control practices was mainly influenced through positive attitudes towards sustainable mange control and the perceived pressure of significant others [29]. The main underlying mechanism for these effects can be explained by the bandwagon heuristic, i.e., the perception that others approve and engage in a specific behaviour. More specifically, the perception that other farmers have a positive attitude towards sustainable mange control influences the farmers’ own attitude and subjective norms and, subsequently, their intention to adopt sustainable practices [29]. This stresses the importance of farmers’ reference groups and the influence these have on treatment decisions.

Furthermore, farmers’ positive attitude towards preventive use of anthelmintics was also identified as a barrier for possible uptake. AR, on the other hand, was not perceived as a risk factor and had no effect on the adoption intention of the dairy farmers. A possible explanation is that current control measures are still perceived to be effective in dairy farms, therefore gaining positive attitudinal support and a low immediate awareness of the risk [48]. Similarly, Ritter and colleagues suggested that low awareness for infectious diseases could be due to a lack of obvious clinical signs or a lack of diagnostic test sensitivity [32]. Gastrointestinal nematode infections in cattle, unless severe, lack obvious clinical signs. In addition, AR is often not associated with obvious clinical signs and there is no routine measurement of drug effectiveness, masking the emergence of AR. It is more challenging to motivate control strategies for diseases that spread silently, particularly because farmers, and humans in general, evaluate any problem in relation to other issues that demand their attention [32].

In a follow-up study, Vande Velde et al. [47] aimed at presenting a more holistic view of farmers’ decision-making in worm control and to identify barriers that prevent farmers from moving from intention towards actual behaviour. Habitual practices, such as routinely applied anthelmintics, blindly following the behaviour of peer farmers, and assigning the responsibility of testing regimes to their veterinarian, were added as probable barriers determining sustainable worm control practices, at least on Belgian dairy farms [47]. Since this follow-up study was based on qualitative data, it did not allow for generalisation of the findings. Therefore, the proposed mechanisms were taken into consideration when developing the conceptual framework for this study.

**Figure 1:**
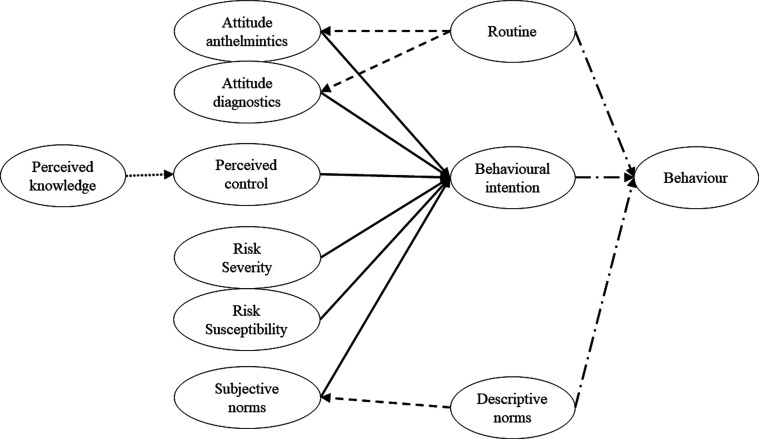
Conceptual model to predict dairy farmers’ adoption of diagnostic methods for control of gastrointestinal nematode infections, based on the work by Vande Velde et al. (2015), and extended with insights from previous decision-making frameworks for parasite control. *Notes.* Full lines represent the equations informed by Vande Velde et al. (2015), dotted lines by Rose Vineer et al. (2017), short-dashed lines by Mingolla et al. (2019), dashed-dotted lines by Vande Velde et al. (2018). The following factors are included: Perceived knowledge (i.e., perception of the current knowledge one has on the context of control of gastrointestinal nematode infections); Perceived control (i.e., perceived ability to adopt diagnostics for control of gastrointestinal nematode infections); Routine (i.e., perception of the current treatment); Attitude diagnostics (i.e., evaluation of the expected outcomes to adopt diagnostics for control of gastrointestinal nematode infections); Attitude anthelmintics (i.e., evaluation of the expected outcomes to preventively treat with anthelmintics for control of gastrointestinal nematode infections); Descriptive norms (i.e., perception of peers that actually perform a diagnosis for control of gastrointestinal nematode infections); Subjective norms (i.e., perception of the expectation of significant others in performing a diagnosis for control of gastrointestinal nematode infections); Perceived susceptibility (i.e., perception of the vulnerability to anthelmintic resistance); Perceived severity (i.e., perception of the impact of anthelmintic resistance); Behavioural intention (i.e., intention to adopt diagnostics for control of gastrointestinal nematode infections); and Behaviour (i.e., self-reported adoption of diagnostics for control of gastrointestinal nematode infections).

The conceptual framework was primarily based on the work of Vande Velde et al. [48] on the use of diagnostics for gastrointestinal nematode control in dairy cattle in Belgium, using a combination of elements of the TPB: attitude (i.e., evaluation of the specific behaviour based on the expected outcomes); subjective norms (i.e., perception of the expectation of significant others in performing the specific behaviour); perceived behavioural control (i.e., perceived ability to perform a specific behaviour) and behavioural intention (i.e., the intention to engage in that specific behaviour), and the HBM: perceived susceptibility (i.e., perception of the vulnerability to the risk); perceived severity (i.e., perception of the impact of the risk); perceived barriers (i.e., perceptions that inhibit the performance of a specific behaviour). Additionally, we added elements found in other studies that measured sustainable parasite control, and which were found to have a significant predictive power. Perceived knowledge (i.e., perception of the current knowledge one has on the context of the specific behaviour) was found to have an indirect effect on adoption intention of faecal worm egg counts (FECs) in horse owners in the UK [36]. Mingolla and colleagues focused on sustainable mange practices on Belgian beef farms [29], and included elements of routine (i.e., perception of the current treatment), referred to as default bias, and descriptive norms (i.e., perception of peers that actually engage in the specific behaviour), referred to as bandwagon bias. Finally, we included self-reported behaviour, to understand the predictive power of behavioural intention and other elements such as routine and descriptive norms [47] on actual behaviour. Figure 1 presents an overview of the conceptual framework. For the purpose of this study, we identified the specific behaviour as the adoption of diagnostics for control of gastrointestinal nematode infections, and AR as the risk.

Due to different cultural and geographical contexts of farmers, it is impossible to extrapolate previous findings from Belgian dairy farmers and provide a general explanation for worm control practices throughout Europe. Regional differences can be expected due to, but not exclusively, a variety in climates [8], AR status [35], or production methods such as grazing management practices [45], which could result in greater infection pressure and, subsequently, increased awareness and risk perception of the disease and AR. Additionally, differences in consumer behaviour [31] or agricultural employment [18] status can change the attitudinal or normative perceptions of a farmer, hence, the intention to change practices. Therefore, this study developed an expanded behavioural framework, based on the insights from previous quantitative and qualitative studies [46], validated the behavioural framework through the European COST Action COMBAR (COMBatting Anthelmintic Resistance in Ruminants; https://www.combar-ca.eu), and identified regional differences amongst the northern, central, and southern parts of Europe. The insights gained through this study increase the understanding of the uptake of diagnostics for sustainable worm control among dairy farmers across Europe.

## Materials and methods

### Multi-lingual survey design

All factors (i.e., elements measured as latent variables) included in the measurement model were built through a set of items (i.e., questions or statements to be evaluated by the participants), which were validated through previous studies [29, 36, 48]. Supplementary file 1 gives a detailed overview of the included factors, corresponding items and sources, measurement scales (7 point bi-polar or Likert scale). Additionally, the survey included socio-demographic questions (location of the farm, age of the farmer and years of experience), farm-specific questions (herd size, pasture management, experience with gastrointestinal nematode infections) and worm control responsibility (veterinarian, farmer or others).

To ensure internal validity and reliability of the survey and corresponding factors, we implemented the method of backtranslation [5]. The survey was developed in English and a minimum of two translation-actions were performed by the participating countries. First, the original English version (V1) was translated into the language of the participating country (V2). Secondly, the latter (V2) was re-translated into English as a control measure (V3). Both translations were performed by two independent translators. Afterwards, both English versions (V1 and V3) were compared for consistency. Ideally, each item (question, statement, measurement scale) was found equal in both versions. If this was not the case, the flagged items were adjusted in a novel version of the foreign language (V2b) and translated into another English version (V3b). V3b was then again compared to the original version V1. This process was repeated until V1 and V3 (abc) were found to be identical.

### Study design and data collection

All countries involved in the European COST Action COMBAR (31 European and three near neighbour countries) were invited to participate in the cross-sectional study, from which nine countries agreed to participate: Austria, Germany, Greece, Italy, Norway, Portugal, Romania, The Netherlands and the United Kingdom (UK). The translation of the surveys was verified centrally, but participant recruitment and data collection were implemented and controlled by each country separately. Germany and Austria implemented the same survey, but used a separate data collection method. Ultimately, five countries were omitted from further analysis due to insufficient sample sizes: Greece, Portugal, Romania, The Netherlands and the UK (See sections “Results” and “Limitations” below). The data collection procedure and population for each dataset included in this study is described per country.

#### Norway

The target population for this research involved Norwegian dairy farmers registered in the Norwegian Dairy Recording System (NDRS). This system is administered by TINE SA, the largest dairy cooperative in Norway. A stratified random sampling approach was used for the selection of farmers within counties. In the randomization process, the questionnaire was sent to every 3rd farmer with a functional e-mail address in the NDRS, in total 2206 dairy farmers. These farmers represented 29% of the 7599 dairy herds in Norway in 2019 (Norwegian Agriculture Agency). Participants received an invitation e-mail containing a link to the survey, using the Enalyzer Survey Solution, a reminder was sent after 9 days. The survey was distributed between February – April 2020. As an incentive, one gift card of NOK 4000 was raffled amongst the participants.

#### Germany

According to the Federal Statistical Office (Statistisches Bundesamt), the target population contained a total of 58,351 dairy farms in May 2020. Due to the German data protection policy, it was not possible to obtain contact information from the dairy farms. Therefore, the recruitment process was not based on random sampling, but self-selection of the farmers. The project was advertised through the websites of the German agricultural gazette “Top agrar” and the dairy gazette “Milchrind” as well as the state control association for milk recording Schleswig-Holstein, the organic food association “Bioland e.V.” and the Bavarian milk control association “Bayerischer Milchpruefring”. The call for participation included a link to the online survey, using Umfrageonline as survey platform, which was accessible from March – August 2020. No incentive was provided.

#### Austria

The target population included all dairy farms in the state of Lower Austria, a total of 4163 farms in 2020 (Agrar Markt Austria). Farms with pasture access, and included in the mailing list of the milk control association (Landeskontrollverband, LKV), were selected to participate (*N* = 1150 farms). Farmers received an invitation letter per e-mail containing a link to the online survey. No reminders were sent. The survey was designed using the platform umfrageonline.com which was available from February to May 2020. As an incentive, a free faecal analysis for the first 100 respondents was offered.

#### Italy

The target population included dairy farmers in selected regions of Italy located in the northern (Lombardy, Veneto and Emilia Romagna), central (Umbria and Lazio) and southern (Campania, Basilicata and Calabria) areas of the country. The total population consisted of 13,586 dairy farms (operations), according to the National Data Bank (NDB) on 31 December 2019. Participants were selected through their veterinarians’ and the Italian Farmers’ Association (http://www.aia.it/aia-website/it/home). The selection was mainly driven by the availability of the veterinarians. A total of 925 participants received a printed questionnaire through their farm veterinarians and received a phone call to inquire about their participation. Interviews were performed by the farm veterinarians during their routine animal health consultancy with the farmers who agreed to participate. No incentive was provided.

### Analysis

Responses were coded in a database using the Statistical Package for the Social Sciences (SPSS, IBM SPSS Statistics version 25.0). Firstly, we evaluated the data sample from each participating country. If the data were incomplete or the sample was below the threshold of 150 participants per country, which would allow us to interpret the results independently, the data were excluded. Secondly, to pool the data from each country into a certain region (northern, central, or southern part of Europe), we performed a Levene’ s test of homogeneity to assess the equality of variance. If an item resulted in unequal variance, this was omitted from further analysis to be able to pool the data sets. The variables that resulted in solely one item due to the pooling procedure were compared against the original latent construct by performing a sample’s *t*-test for comparing means, and Levene’s test of homogeneity for comparing variance. In the behavioural sciences, most phenomena of interest are not directly observable and thus are measured with error, hence the use of multi-item scales. Therefore, if the analysis of the sample’s t-test and Levene’s test resulted in a significant difference, the item, hence the variable, was omitted from further analysis. Finally, we assessed the possibility for measuring the full conceptual model, based on sample size requirements [51]. One size does not fit all, and there is great variability in sample size requirements depending on several aspects of the structural equation model (e.g., factor loadings, size of the equations, number of items included in the latent variables). Therefore, we evaluated the sample size along with the evaluation of our models and, if the requirements were not obtained, the models were stripped down to an adequate size.

Structural equation modelling (SEM) was applied to estimate the effect of the psychological factors on the different behavioural techniques. SEM was performed using the lavaan package [38] in the statistical software R (lavaan version 0.6-7, R version 3.5.2, The R Foundation for Statistical Computing, 2016). The maximum likelihood estimation was used to assess missing values, using the Yuan-Bentler correction. Firstly, we inspected the model to detect irregularities in the observed data, such as unsuitable factor loadings (< 0.50, > 1.00) and insignificant variances, and excluded the item if necessary. Afterwards, the equations were evaluated and model fit acquired using the following indices: the Comparative Fit Index (CFI) and the Tucker Lewis Index (TLI) (CFI/TLI > 0.90), the Root Mean Square of Approximation (RMSEA) (<0.08) and the Standard Root Mean Square Residual (SRMR) (<0.10) [19]. Once the model presented a good fit, a multigroup analysis was planned between the three above mentioned regions, based on a methodology described by Varni et al. [49]. The multigroup analysis would allow us to statistically compare models between the three groups.

## Results

### Summary of the responses

Five countries initiated the survey but obtained insufficient responses to allow for rigorous statistical analysis and were therefore omitted: UK (*n* = 11), The Netherlands (*n* = 9), Romania (*n* = 41), Portugal (*n* = 50) and Greece (*n* = 10). Italy recruited a total of 302 participants (32.6% response rate) and was included in the analysis, as well as Norway with 533 participants (24% response rate), and Austria (*n* = 161, 13.9% response rate) and Germany (*n* = 176, no calculated response rate due to self-selection) combined. The data sets were evaluated for incomplete responses and resulted in a *status-quo* for Italy, 507 fully completed surveys for Norway, 133 for Germany and 134 for Austria. Next, both data sets (Germany and Austria) were compared for variance equality prior to pooling. Supplementary file 2 presents the Levene’s test for all items included in the model. Levene’s test showed unequal variance for the following items: Q10_1 (*F*(1, 248) 8.36, *p* = 0.004) corresponding to attitude towards anthelmintics; Q11_1 (*F*(1, 262) 21.92, *p* < 0.001), Q11_2 (*F*(1, 260) 7.85, *p* = 0.005), Q11_3 (*F*(1, 258) 26.42, *p* < 0.001) self-reported behaviour; Q13_2 (*F*(1, 260) 11.95, *p* = 0.001) perceived control; Q15_2 (*F*(1, 260) 4.43, *p* = 0.036), Q15_3 (*F*(1, 261) 4.35, *p* = 0.038) behavioural intention; Q16_3 (*F*(1, 261) 4.67, *p* = 0.032) descriptive norms; Q17_3 (*F*(1, 254) 5.1, *p* = 0.045), Q17_5 (*F*(1, 254) 8.54, *p* = 0.004) perceived knowledge; Q19_5 (*F*(1, 239) 5.84, *p* = 0.016) risk severity. These items were omitted from the analysis, and data from Germany and Austria were pooled into one dataset. Behavioural intention (Q15_1 + Q15_2 + Q15_3) and descriptive norms (Q16_1 + Q16_3) resulted both in a one-item measurement, Q15_1 and Q16_1, respectively. Both items were compared against their corresponding latent construct. No significant difference was detected for Q15_1 compared to behavioural intention (Q15_1 + Q15_2 + Q15_3) for both the *t*-test (*F*(1, 259) −0.76, *p* = 0.45), and the Levene’s test (*F*(1, 521) 0.075, *p* = 0.79). Equally, no significant difference was found for Q16_1 compared to descriptive norms (Q16_1 + Q16_3) for both the *t*-test (*F*(1, 261) 1.365, *p* = 0.173), and the Levene’s test (*F*(1, 520) 1.014, *p* = 0.314). This resulted in the inclusion of Q15_1, representing behavioural intentions, and the inclusion of Q16_1, representing descriptive norms.

### Structural equation models

**Figure 2:**
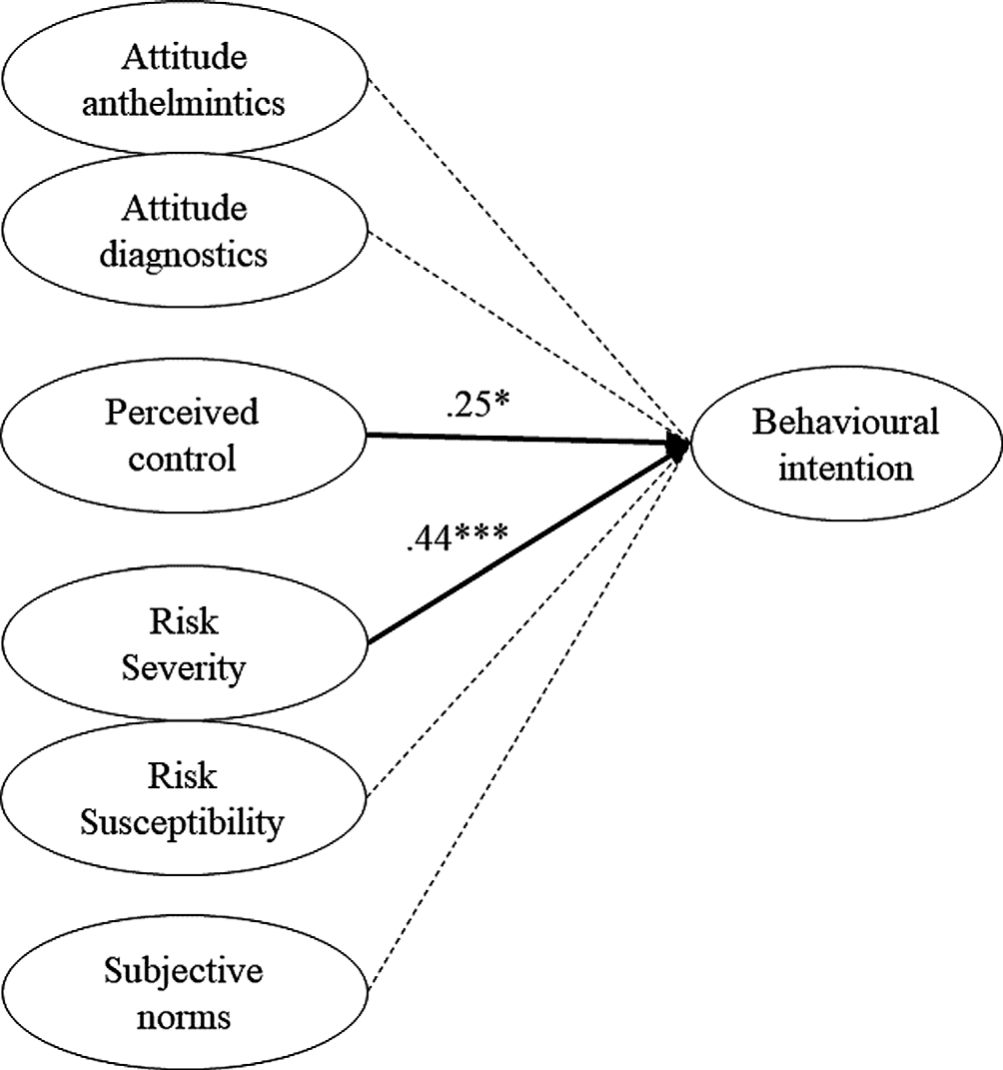
Structural equation model predicting dairy farmers’ adoption of diagnostic methods for control of gastrointestinal nematode infections in Italy (N = 302). *Notes.* Full lines represent the significant equations with the numbers representing standardized regression coefficients; dotted lines represent insignificant equations. ***p < 0.001, **p < 0.01, *p < 0.05.

*Italy*. Due to the limited sample size, we were unable to measure the full conceptual model. Therefore, it was decided to exclude the equations beyond behavioural intention, represented by the dashed-dotted lines in Figure 1. This would allow us to compare the model with former published work [48]. The model was inspected in two separate rounds and results are presented in Supplementary file 3, Section 1, showing the included factor loadings. The following items/variables were excluded from the model due to unsuitable factor loadings (<0.50): Q16_1 + Q16_3 (descriptive norms); Q16_5 (item corresponding to subjective norms); Q13_1 (perceived control); Q17_6 (perceived knowledge). Subsequently, the variables perceived knowledge and routine were excluded, due to low regressions and mediocre factor loadings with a low number of items included [51]. This resulted in a good model fit: CFI = 0.98, TLI = 0.97, RMSEA = 0.035, SRMR = 0.043, Yuan-Bentler = 1.24. The model is represented in Figure 2 and explained 0.42 of the variance in behavioural intention. Risk severity (*β* = 0.44, *z* = 5.34, *p* < 0.001) had the strongest positive effect on behavioural intention, followed by perceived control (*β* = 0.25, *z* = 2.14, *p* < 0.05). Both attitude towards anthelmintics (*β* = 0.00, *z* = 0.00, *p* = 0.99), and attitude towards diagnostics (*β* = −0.012, z = −0.19, *p* = 0.85) had an insignificant effect on behavioural intention, as well as subjective norms (β = 0.22, z = 1.57, *p* = 0.12) and risk susceptibility (*β* = −0.049, *z* = −0.71, *p* = 0.48).

**Figure 3:**
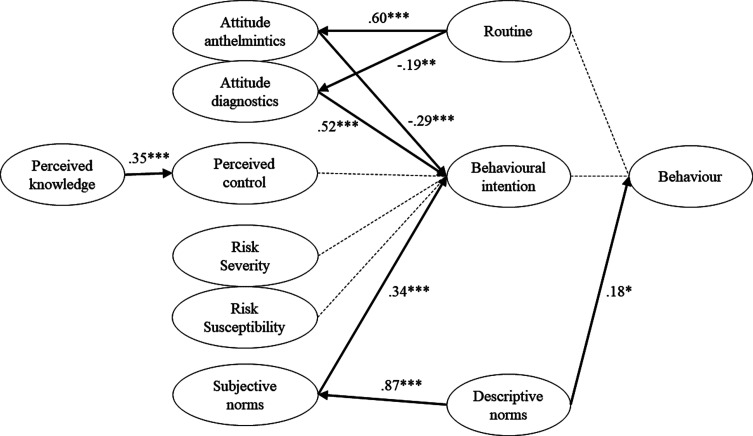
Structural equation model predicting dairy farmers’ adoption of diagnostic methods for control of gastrointestinal nematode infections in Norway (N = 507). *Notes.* Full lines represent the significant equations with the numbers representing standardized regression coefficients; dotted lines represent insignificant equations. ***p < 0.001, **p < 0.01, *p < 0.05.

*Norway*. Due to a larger sample size, we were able to evaluate the full conceptual model. The model was inspected in two separate rounds and results are presented in Supplementary file 3, Section 2. The following items were excluded from the model due to unsuitable factor loadings (<0.50): Q10_3 (item corresponding to attitudes towards anthelmintics); Q13_1 (perceived control); Q17_3 + Q17_5 + Q17_6 (perceived knowledge). This resulted in a good model fit: CFI = 0.95, TLI = 0.94, RMSEA = 0.048, SRMR = 0.086, Yuan-Bentler = 1.12. The model is represented in Figure 3 and explained 0.43 of the variance in behavioural intention and 0.96 of the variance in behaviour. Attitude towards diagnostics positively affected behavioural intention (*β* = 0.52, *z* = 9.89, *p* < 0.001), as did the variable subjective norms (*β* = 0.34, *z* = 5.93, *p* < 0.001). In contrast, attitude towards anthelmintics correlated negatively with behavioural intention (*β* = −0.29, z = −4.88, *p* < 0.001). Other variables were found to have no significant effect on behavioural intention: perceived control (*β* = −0.067, *z* = −1.41, *p* = 0.15), perceived severity (*β* = 0.042, *z* = 0.844, *p* = 0.4) and perceived susceptibility (*β* = 0.017, z = 0.35, *p* = 0.73). Additionally, routine had a positive effect on attitude towards anthelmintics (*β* = 0.60, z = 6.89, *p* < 0.001), but a negative effect on attitude towards diagnostics (*β* = −0.19, *z* = −2.87, *p* < 0.01). Descriptive norms were found to positively correlate with subjective norms (*β* = 0.87, *z* = 13.05, *p* < 0.001), and perceived knowledge with perceived control (*β* = 0.35, *z* = 5.6, *p* < 0.001). Finally, self-reported behaviour was merely positively affected by descriptive norms (*β* = 0.18, *z* = 2.31, *p* < 0.05), whereas routine (*β* = −0.076, *z* = −0.95, *p* = 0.34) and behavioural intention (*β* = −0.029, *z* = −0.34, *p* = 0.74) had no significant effect.

**Figure 4:**
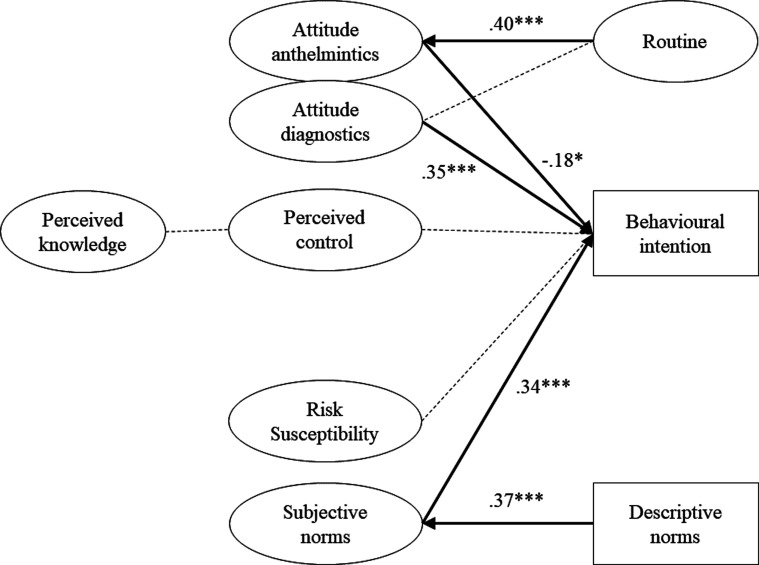
Structural equation model predicting dairy farmers’ adoption of diagnostic methods for control of gastrointestinal nematode infections in Austria and Germany (N = 267). *Notes.* Full lines represent the significant equations with the numbers representing standardized regression coefficients, dotted lines represent insignificant equations. ***p < 0.001, **p < 0.01, *p < 0.05. Variables represented by a rectangle consist of a one-item measurement; variables represented by an oval consist of a multi-item measurement.

*Austria and Germany*. Due to a smaller sample size and restrictions due to pooling of the data such as excluding self-reported behaviours, it was impossible to evaluate the full conceptual model. The model was inspected in one round and results are presented in Supplementary file 3, Section 3. The following item was excluded from the model due to a negative variance: Q19_1 (item corresponding to perceived severity). Severity resulted in a one-item measurement, and therefore needed to be compared against its corresponding latent construct. A significant difference was found for Q19_1 compared to perceived severity (Q19_1 + Q19_3) for both the *t*-test (*F*(1, 259) −5.14, *p* < 0.001), and the Levene’s test (*F*(1, 519) 7.87, *p* = 0.005). Hence, we excluded Q19_1, representing risk severity. This resulted in a good model fit: CFI = 0.92, TLI = 0.91, RMSEA = 0.061, SRMR = 0.095, Yuan-Bentler = 1.06. The model is represented in Figure 4 and explained 0.65 of the variance in behavioural intention. Attitude towards diagnostics positively affected behavioural intention (*β* = 0.35, *z* = 3.67, *p* < 0.001), as did the variable subjective norms (*β* = 0.34, *z* = 4.11, *p* < 0.001). In contrast, attitude towards anthelmintics correlated negatively with behavioural intention (*β* = −0.18, *z* = −1.96, *p* = 0.05). Other variables were found to have no significant effect on behavioural intention: perceived control (*β* = 0.14, *z* = 1.46, *p* = 0.14) and perceived susceptibility (*β* = 0.023, *z* = 0.30, *p* = 0.77). Additionally, routine had a positive effect on attitude towards anthelmintics (*β* = 0.40, *z* = 4.46, *p* < 0.001), but no significant effect on attitude towards diagnostics (*β* = −0.12, *z* = −1.15, *p* = 0.25). Descriptive norms had a positive effect on subjective norms (*β* = 0.37, *z* = 3.45, *p* = 0.001). Perceived knowledge and perceived control showed no significant correlation (*β* = 0.44, *z* = 1.06, *p* = 0.29).

### Multi-group analysis

Due to the differences in the data sets resulting from data pooling, as well as the exclusion of certain items and variables resulting from unsuitable factor loadings and negative variances, a suitable model that was comparable among the different populations was not found. Hence, a multi-group analysis could not be performed, and no statistical inferences could be made across the three models.

## Discussion

The uptake of sustainable worm control practices is becoming increasingly important, considering the growing threat of AR in European ruminant farming systems, including dairy cattle [35]. To understand farmers’ decision-making for control of gastrointestinal nematode infections, and the elements contributing to the uptake of diagnostic methods, a multi-country cross-sectional study was performed with the aim of developing and validating a general behavioural framework and to compare the elements amongst the different populations. However, due to the cultural and contextual differences in the included populations, leading to significant variations in the responses provided, we were unable to perform this multi-group analysis. Nevertheless, we were able to measure three distinct models, representing three distinct regions, that allow for narrative rather than statistical comparison.

The models representing Norway, Austria and Germany were found to be the most similar, and in line with previous research in parasite control [28, 36, 48], or more generally, with strategies for infectious disease prevention and control [32]. A positive attitude towards diagnostic methods indicates a positive adoption intention, in contrast, a positive attitude towards anthelmintics was associated with a decreased intention for adopting diagnostics. Both elements were affected by farmers’ satisfaction of their current control measures, hence, their routine practices. Routine had a positive effect on attitude towards anthelmintics, but a negative effect on attitudes towards diagnostics. Because the use of anthelmintics without prior diagnosis is the current default practice in many countries, this result indicates that the current control measures are still perceived to be effective on dairy farms. Additionally, this may explain why the perception of severity and susceptibility of the threat of AR had no direct effect on farmers’ intention to adopt diagnostics. In contrast, AR is considered a serious problem in sheep; therefore, risk severity and susceptibility did have a significant effect on sheep farmers’ behaviour intention in a previous study by Jack and colleagues [20].

The effect of the farmers’ community (i.e., descriptive norms) was evaluated indirectly through the effect of subjective norms on behavioural intention, or directly on behaviour. Both had a positive effect, which contrasted with Vande Velde et al. [47] who found peers as subjective norms to have no effect on behaviour intention, since their opinion was not valued. However, this result is in line with a review about antimicrobial use on dairy farms, suggesting a positive effect of social pressure through both internal motivation, represented in our models by the indirect route, and external motivation, represented by the direct route [26]. Our study presents empirical validation of these two routes through the Norwegian dairy farmer population. In addition, our results presented a dominant effect of the external route compared to the “insignificant” internal route through behavioural intentions. This indicates that a direct, perhaps unconscious, effect of community is stronger than internal motivations. However, due to the ambiguity of measuring behaviour through a self-reporting scale [16], we interpret our results as a trend rather than assuming no effect of behavioural intention on behaviour.

The model representing Italy differs greatly from what is known when using socio-psychological models for understanding farmers’ behaviour in parasite control. The results represent a behavioural intention that is driven by a perception of risk, similar to farmers in a context of emergency such as drought [25]. The protection motivation theory [34] explains human behaviour in a context of risk, and includes two main factors: threat appraisal and self-efficacy, which is somewhat similar to our elements of risk perception and perceived behavioural control. This result indicates that farmers in southern Europe consider AR a severe threat and are willing to act on it. Although AR has not been reported as a major problem in Italy [4, 13, 35], risk perception is known to increase, among other things, with the novelty of the risk [40]. Potentially, this could explain the insignificant effects of the other elements such as attitudes and subjective norms on behavioural intention. If knowledge lacks on a probable issue, opinions will not readily be formed. Hence, non-existing opinions, by the farmer or its subjective norms, are not able to influence behavioural intention. Nevertheless, we believe this discrepancy could be due to a different collection method. The farm veterinarian collecting responses during a routine animal health consultancy could have instigated some form of social control. This social control can result in presenting socially desirable answers, as well as reactance due to forced responses [39]. To some extent, this model should be interpreted as a trend rather than assuming no effect of the other elements included in the framework.

## Limitations

Although we aimed to cluster the results into three regions: northern, central, and southern Europe, the results should not be interpreted as being representative of these regions. Our intention was to differentiate between distinct groups, based on geography and climate, and presumably epidemiological traits [7] of worm infections. We acknowledge that our number of samples is limited and not suitable to make these assumptions. At the same time, there will be significant differences between countries in the same region due to regulations, farming styles and social context. Therefore, we implemented a threshold of 150 participants, to allow for independent interpretation of the results per country. Consequently, we were unable to include the data from participating countries with a sample size below the threshold, which results in a loss of information. In retrospect, a sample size of 150 participants was also deemed unsuitable for the measurement and interpretation of the conceptual model [51], which resulted in pooling the Austrian and German datasets. Concerning the unsuccessful cross-country comparison, the back-translation methodology appeared to be unsuitable for multi-group analysis, especially when measuring personal values and opinions for such a diverse population [2]. Additionally, the many nuances in between languages itself make it difficult to parametrise conceptual elements [52]. Not only language nuances, but cultural differences between farmers who share the same language but come from different regions appeared to have a stronger effect than initially anticipated, hence the limitations in pooling the Austrian and German datasets. Finally, different collection methods, such as providing distinct incentives (e.g., monetary lotteries of 4000 NOK vs. free services in diagnosis for a number of participants), draws a distinct group of participants, resulting in unmatched datasets and different interpretations of the outcomes. Therefore, we strongly recommend taking the following measures into account when aiming for a multi-group comparison between farmers: (1) understanding and considering contextual and cultural differences by performing formative research before initiating the survey (i.e., collecting information by reviewing the literature on regional differences within agricultural practices, as well as performing in-depth interviews to inform the development of the survey); (2) more rigorous piloting of the materials should be implemented if the aim is to make statistical comparisons between groups (i.e., the surveys should be pre-tested in each participating country, compared, and adapted for equal variance of the responses); (3) the data collection procedure should be managed centrally to obtain uniformity amongst the population and datasets (i.e., the surveys should be collected from one platform only, using the same distribution method and rewarding the same incentive). Finally, the population included in the study possibly represents more engaged farmers, and results should be interpreted as such. However, more engaged farmers are known to show more tendency to change behaviour and to adapt to new practices and should therefore be targeted in any first attempt at making substantial changes throughout the farming community [14].

## Conclusions

This study was able to capture the adoption potential of diagnostics as sustainable control of gastrointestinal nematodes for three distinct regions in Europe. Our results point to the need for separate actions within Europe to promote the use of diagnostics. In Norway, Austria and Germany, two actions are recommended to change the course of AR. Firstly, to discourage and disrupt their current routines by implementing targeted communication messages and more subtle behaviour change techniques such as nudging [17]. Nudging is a technique that changes the environment in such a way that people will engage in the encouraged behaviour without denying them a choice, and is specifically useful for changing habits and routines [50]. These techniques should work complementarily to eventually change the farmers’ attitudes and behaviours in the long run. Secondly, the importance of farmers’ peers has yet again been highlighted and should therefore be included in any action to change farmer behaviours. The identification of innovators and early adaptors to disseminate ideas and messages, that could instigate a ripple effect of change, should be prioritized in any communication strategy. Any such action should be evaluated for its impact first, and afterwards scaled up towards the whole sector. In Italy, results indicate a need to target communication messages that focus on the threat of AR, and at the same time present behavioural actions to control this threat, hence the adoption of diagnostics. Nevertheless, to truly understand the nature of these surprising findings, a more comprehensive long-term study should be performed to study this discrepancy in more depth. Finally, this study identified several bottlenecks of multi-country comparisons and highlights the importance of formative research, rigorous piloting and centralised data collection.

## Supplementary Materials

Supplementary materials are available at https://www.parasitejournal.org/10.1051/parasite/2023002/olm. *Supplementary file 1*: The factors included in the measurement model, corresponding items, labels and measurement scales. *Supplementary file 2*: Test of homogeneity of variance through Levene’s test analysis. *Supplementary file 3*: Sections 1, 2, 3
