# Supplementary material for: Understanding the uptake of diagnostics for sustainable gastrointestinal nematode control by European dairy cattle farmers: a multi-country cross-sectional study
Source: Parasite. 2023 Feb 10;30:4. doi: 10.1051/parasite/2023002 (PMC9912928; doi:10.1051/parasite/2023002)
Supplement: Supplementary file 1 — The factors included in the measurement model, corresponding items, labels and measurement scales. [file parasite-30-4-s1.pdf]

Supplementary file 1

The factors included in the measurement model, corresponding items, labels and measurement scales.

Factor label	Item label	Item statement	Item measurement
Routine	Q8_1	I always apply the same treatment	7-point Likert scale[a]
	Q8_2	I do not feel the need to change my current treatment	7-point Likert scale[a]
	Q8_3	The treatment I currently apply, is the default treatment I always apply	7-point Likert scale[a]
Attitude anthelmintics	Q10_1	In my opinion, the application of dewormers to prevent worm infections on my farm is…	7-point bipolar scale[b]
	Q10_2	In my opinion, the application of dewormers to prevent worm infections on my farm is…	7-point bipolar scale[c]
	Q10_3	In my opinion, the application of dewormers to prevent worm infections on my farm is…	7-point bipolar scale[d]
Behaviour	Q11_1	Do you use some form of diagnosis to detect worm infections on your farm? – Faecal test	5-point Likert scale[e]
	Q11_2	Do you use some form of diagnosis to detect worm infections on your farm? – Bulk-milk test	5-point Likert scale[e]
	Q11_3	Do you use some form of diagnosis to detect worm infections on your farm? – Blood test	5-point Likert scale[e]
Perceived control	Q13_1	The decision to use worm diagnosis on my farm is under my control	7-point Likert scale[a]
	Q13_2	I can easily command for a diagnosis on my farm	7-point Likert scale[a]
	Q13_3	I can have a diagnosis whenever I want	7-point Likert scale[a]
Attitude diagnostics	Q14_1	In my opinion, to diagnose before treating my animals with dewormers is...	7-point bipolar scale[b]
	Q14_2	In my opinion, to diagnose before treating my animals with dewormers is...	7-point bipolar scale[c]
	Q14_3	In my opinion, to diagnose before treating my animals with dewormers is...	7-point bipolar scale[d]
Behavioural intention	Q15_1	I expect to diagnose before using dewormers in the future	7-point Likert scale[a]
	Q15_2	I want to diagnose before using dewormers preventively on my farm	7-point Likert scale[a]
	Q15_3	I intent to diagnose before treating my animals with dewormers	7-point Likert scale[a]
Descriptive norms	Q16_1	Most farmers use a diagnosis before deworming on their farms	7-point Likert scale[a]
	Q16_3	Most of my colleagues diagnose before they preventively apply dewormers	7-point Likert scale[a]
Subjective norms	Q16_2	People who are important to me think that I should diagnose before using dewormers on my farm	7-point Likert scale[a]
	Q16_4	It is expected of me that I diagnose before treatment	7-point Likert scale[a]
	Q16_5	I feel social pressure to diagnose before using dewormers on my farm	7-point Likert scale[a]

Perceived knowledge	Q17_1	I currently know enough about worms and worming to decide on an appropriate worm control programme on my farm	7-point Likert scale[a]
	Q17_2	I have enough information available to me that I can use to decide on an appropriate worm control programme on my farm	7-point Likert scale[a]
	Q17_3	I know what worm diagnostics are for	7-point Likert scale[a]
	Q17_4	I know the limitations of worm diagnostics (i.e. what they can't tell you)	7-point Likert scale[a]
	Q17_5	I can interpret the results of a diagnosis and act on the information with the help of a vet/adviser	7-point Likert scale[a]
	Q17_6	I can interpret the results of a diagnosis and act on the information on my own	7-point Likert scale[a]
Risk severity	Q19_1	Anthelmintic resistance is a severe threat for animal health	7-point Likert scale[a]
	Q19_3	Anthelmintic resistance is a serious threat for animal health	7-point Likert scale[a]
	Q19_5*	Anthelmintic resistance is a significant threat for animal health	7-point Likert scale[a]
Risk susceptibility	Q19_2	It is likely that wormson my farm will develop anthelmintic resistance	7-point Likert scale[a]
	Q19_4	The worms on my farm are at risk to develop anthelmintic resistance	7-point Likert scale[a]
	Q19_6*	It is probable that the worms on my farm will develop anthelmintic resistance	7-point Likert scale[a]

[a] 1= Strongly disagree; 2 = Disagree; 3 = Slightly disagree; 4 = Neutral; 5 = Slightly agree; 6 = Agree; 7 = Strongly agree

[b] 1 = Bad – 7 = Good

[c] 1 = Useless – 7 = Useful

[d] 1 = Harmful – 7 = Beneficial

[e] 1 = Never; 2 = I've tried it once; 3 = I've tried it more than once; 4 = When I sense problems; 5 = Every year

* Not included in the Norwegian survey due to unsuitable translation
