# Supplementary material for: Understanding the uptake of diagnostics for sustainable gastrointestinal nematode control by European dairy cattle farmers: a multi-country cross-sectional study
Source: Parasite. 2023 Feb 10;30:4. doi: 10.1051/parasite/2023002 (PMC9912928; doi:10.1051/parasite/2023002)
Supplement: Supplementary file 2 — Test of homogeneity of variance through Levene’s test analysis. [file parasite-30-4-s2.pdf]

Supplementary file 2

Test of homogeneity of variance through Levene's test analysis

Factor label	Item label	BF Statistic *F*	df1	df2	Sig. *p*
Routine	Q8_1	.801	1	259	.372
	Q8_2	.003	1	258	.957
	Q8_3	1.962	1	257	.162
Attitude anthelmintics	Q10_1	8.355	1	248	.004*
	Q10_2	3.647	1	248	.057
	Q10_3	.041	1	244	.840
Behaviour	Q11_1	21.915	1	262	.000*
	Q11_2	7.847	1	260	.005*
	Q11_3	26.420	1	258	.000*
Perceived control	Q13_1	2.782	1	263	.097
	Q13_2	11.950	1	260	.001*
	Q13_3	.083	1	261	.773
Attitude diagnostics	Q14_1	.333	1	255	.564
	Q14_2	.3640	1	257	.058
	Q14_3	1.006	1	251	.317
Behavioural intention	Q15_1	3.266	1	262	.072
	Q15_2	4.427	1	260	.036*
	Q15_3	4.350	1	261	.038*
Descriptive norms	Q16_1	2.195	1	259	.140
	Q16_3	4.674	1	261	.032*
Subjective norms	Q16_2	.894	1	259	.345
	Q16_4	.996	1	260	.319
	Q16_5	1.952	1	261	.164
Perceived knowledge	Q17_1	.195	1	256	.659
	Q17_2	.025	1	255	.875
	Q17_3	4.050	1	254	.045*
	Q17_4	.336	1	254	.563
	Q17_5	8.542	1	254	.004*
	Q17_6	.218	1	254	.641
Risk severity	Q19_1	3.356	1	243	.068
	Q19_3	3.641	1	244	.058
	Q19_5	5.844	1	239	.016*
Risk susceptibility	Q19_2	.180	1	244	.672
	Q19_4	.877	1	243	.350
	Q19_6	.097	1	240	.755

* unequal variance is established ($p < .05$)
