# Supplementary material for: Understanding the uptake of diagnostics for sustainable gastrointestinal nematode control by European dairy cattle farmers: a multi-country cross-sectional study
Source: Parasite. 2023 Feb 10;30:4. doi: 10.1051/parasite/2023002 (PMC9912928; doi:10.1051/parasite/2023002)
Supplement: Supplementary file 3 — Sections 1, 2, 3 [file parasite-30-4-s3.pdf]

Supplementary file 3
Section 1

Structural equation modelling of the dataset Italy.

Factor loadings of the items included in the primary model

Factor label	Item label	Factor estimate	Std. error	Std. loading
Routine	Q8_1	1.000		.524[b]
	Q8_2	1.003	.288	.800[b]
	Q8_3	1.393	.288	.692[b]
Attitude anthelmintics	Q10_1	1.000		.793
	Q10_2	1.019	.179	.586
	Q10_3	1.041	.222	.562
Perceived control	Q13_1	1.000		.455[a]
	Q13_2	1.565	.171	.929
	Q13_3	1.449	.158	.834
Attitude diagnostics	Q14_1	1.000		.812
	Q14_2	.914	.103	.726
	Q14_3	.894	.092	.726
Behavioural intention	Q15_1	1.000		.841
	Q15_2	.995	.048	.793
	Q15_3	.992	.059	.856
Descriptive norms	Q16_1	1.000		.397[a]
	Q16_3	1.777	.242	.785[a]
Subjective norms	Q16_2	1.000		.585
	Q16_4	1.012	.123	.605
	Q16_5	.656	.155	.408[a]
Perceived knowledge	Q17_1	1.000		.751[b]
	Q17_2	.950	.068	.721[b]
	Q17_3	.470	.120	.350[a]
	Q17_4	.635	.108	.454[b]
	Q17_5	.461	.133	.327[a]
	Q17_6	.479	.112	.335[a]
Risk severity	Q19_1	1.000		.840
	Q19_3	.996	.057	.870
	Q19_5	.942	.069	.834
Risk susceptibility	Q19_2	1.000		.794
	Q19_4	1.006	.077	.824
	Q19_6	1.084	.090	.833

[a] items/variables excluded from further analysis due to unsuitable factor loadings (< 0.50, > 1.00) during round 1

[b] items/variables excluded from further analysis due to due unsuitable regressions (e.g., unsignificant correlations) and unstable construct measurements (e.g., cross-factorial correlations) during round 2

Section 2

Structural equation modelling of the dataset Norway.

Factor loadings of the items included in the primary model

Factor label	Item label	Factor estimate	Std. error	Std. loading
Routine	Q8_1	1.000		.838
	Q8_2	.671	.064	.617
	Q8_3	.932	.039	.925
Attitude anthelmintics	Q10_1	1.000		.751
	Q10_2	1.246	.138	.804
	Q10_3	.829	.128	.485[a]
Behaviour	Q11_1	1.000		.899
	Q11_2	.962	.172	.603
	Q11_3	1.162	.222	.834
Perceived control	Q13_1	1.000		.419[a]
	Q13_2	2.663	.350	.938
	Q13_3	2.432	.359	.873
Attitude diagnostics	Q14_1	1.000		.857
	Q14_2	1.123	.056	.932
	Q14_3	.833	.065	.696
Behavioural intention	Q15_1	1.000		.899
	Q15_2	1.051	.040	.931
	Q15_3	1.066	.035	.938
Descriptive norms	Q16_1	1.000		.859
	Q16_3	.891	.058	.865
Subjective norms	Q16_2	1.000		.792
	Q16_4	1.119	.078	.860
	Q16_5	0.797	.075	.644
Perceived knowledge	Q17_1	1.000		.883
	Q17_2	.958	.026	.922
	Q17_3	.497	.092	.463[a]
	Q17_4	.563	.075	.597
	Q17_5	.544	.089	.498[a]
	Q17_6	.497	.083	.520[b]
Risk severity	Q19_1	1.000		.959
	Q19_3	.771	.196	.723
Risk susceptibility	Q19_2	1.000		.959
	Q19_4	.952	.158	.847

[a] items excluded from further analysis due to unsuitable factor loadings (< 0.50, > 1.00) during round 1

[b] items excluded from further analysis due to unsuitable factor loadings (< 0.50, > 1.00) during round 2

Section 3

Structural equation modelling of the pooled dataset Austria and Germany.

Factor loadings of the items included in the primary model

Factor label	Item label	Factor estimate	Std. error	Std. loading
Routine	Q8_1	1.000		.777
	Q8_2	.720	.088	.580
	Q8_3	1.013	.087	.862
Attitude anthelmintics	Q10_2	1.000		.934
	Q10_3	.932	.147	.845
Perceived control	Q13_1	1.000		.511
	Q13_3	2.077	1.447	.881
Attitude diagnostics	Q14_1	1.000		.818
	Q14_2	1.279	.106	.941
	Q14_3	.976	.097	.824
Subjective norms	Q16_2	1.000		.700
	Q16_4	1.282	.204	.830
	Q16_5	0.841	.159	.582
Perceived knowledge	Q17_1	1.000		.901
	Q17_2	.950	.046	.878
	Q17_4	.699	.074	.655
	Q17_5	.682	.077	.632
Risk severity	Q19_1	1.000		.695[a]
	Q19_3	1.455	.913	1.056[a]
Risk susceptibility	Q19_2	1.000		.795
	Q19_4	1.199	.111	.859
	Q19_6	1.365	.119	.940

[a] items/variables excluded from further analysis due to unsuitable factor loadings (< 0.50, > 1.00).
